# Cognitive Factors of Using Health Apps: Systematic Analysis of Relationships Among Health Consciousness, Health Information Orientation, eHealth Literacy, and Health App Use Efficacy

**DOI:** 10.2196/jmir.3283

**Published:** 2014-05-09

**Authors:** Jaehee Cho, Dongjin Park, H Erin Lee

**Affiliations:** ^1^School of Mass CommunicationChung-Ang UniversitySeoulRepublic of Korea; ^2^School of CommunicationHallym UniversityChuncheon, Gangwon-doRepublic of Korea; ^3^Media Communication DivisionHankuk University of Foreign StudiesSeoulRepublic of Korea

**Keywords:** health apps, health consciousness, health information orientation, eHealth literacy, health app use efficacy

## Abstract

**Background:**

Interest in smartphone health apps has been increasing recently. However, we have little understanding of the cognitive and motivational factors that influence the extent of health-app use.

**Objective:**

This study aimed to examine the effects of four cognitive factors—health consciousness, health information orientation, eHealth literacy, and health-app use efficacy—on the extent of health-app use. It also explored the influence of two different use patterns—information and information-behavior use of health apps—with regard to the relationships among the main study variables.

**Methods:**

We collected and analyzed 765 surveys in South Korea. According to the results, there was a negligible gender difference: males (50.6%, 387/765) and females (49.4%, 378/765). All participants were adults whose ages ranged from 19 to 59. In order to test the proposed hypotheses, we used a path analysis as a specific form of structural equation modeling.

**Results:**

Through a path analysis, we discovered that individuals’ health consciousness had a direct effect on their use of health apps. However, unlike the initial expectations, the effects of health information orientation and eHealth literacy on health-app use were mediated by health-app use efficacy.

**Conclusions:**

The results from the path analysis addressed a significant direct effect of health consciousness as well as strong mediating effects of health-app use efficacy. These findings contribute to widening our comprehension of the new, digital dimensions of health management, particularly those revolving around mobile technology.

## Introduction

### Background

Recently, along with the notable development of mobile communication devices, mobile health, known as mHealth, has become one of the hottest issues in the disciplines of medical science, nursing, and health communication [1-3]. The main factors leading this mobile health boom have been the high penetration of Internet access, particularly expanding Wi-Fi-services, continuous improvement of mobile supporting systems, and increased use of smartphones. For instance, 91% of US adults now own cellular phones [4], and 53% of cell phone users (45% of all US adults) own a smartphone [5]. Supported by these advanced mobile service and technological developments, people have come to actively seek health information through the Internet using their mobile devices [5]. For example, 52% of smartphone users seek health information through their smart devices [5].

In addition to such information-seeking behaviors through smart devices, there has been a notable increase of health apps available on such devices [3,5-12]. According to Kamerow [13], there are approximately 100,000 health-related apps for smartphones; by 2015, about 500 million owners of smartphones throughout the world will use health apps. In 2012, while 84% of smartphone users had downloaded at least one app, 19% of them had used a health app for the purpose of tracking and managing their health [5]. According to the authors, the adoption of health apps was stronger among females, young populations, and people with higher income. These apps provide users with health-related services, such as medical information, blood pressure checks, female health checks, and so on. With regard to the specific functions of health apps, there are three dominant areas: exercise, diet, and weight management [5]. For example, 38% of health-app users use a health app in order to track their exercise [5].

As noted above, the fact that there has been a great increase of health apps on smart devices can hardly be denied [3,5-12]. Thus, recently, previous studies in the areas of medical science and informatics have intensively investigated the effectiveness of specific apps on smartphones [3,5-12]. In particular, they have focused on scrutinizing those functions and features of smartphone apps that are specialized for particular health conditions (eg, obesity) or diseases (eg, diabetes). For example, Morris et al evaluated health applications on cellular phones, particularly for emotional self-awareness [9]. Kirwan et al and Frøisland et al paid significant attention to smartphone apps for Type 1 diabetes [11-12]. These studies meaningfully contributed to widening our understanding of the effectiveness of interventions of particular smartphone apps.

Nevertheless, we have little understanding of the general cognitive motivators that trigger people’s use of health apps, which are relevant to individuals’ personal psychological conditions. Except for Lim et al’s study [14], there has been little empirical research on the cognitive motivational factors of health-app use in general. Without proper comprehension of the motivational and cognitive factors of adopting and using health apps, it would be difficult to fully understand individuals’ use of such apps. Hence, this present study aimed at exploring which cognitive factors would lead people to use health apps among smartphone owners in South Korea. This particular country is adequate for the present study due to its high Internet penetration rate as well as its high distribution rate of smartphones. According to the 2013 Internet Use Report [15], the Internet use rate among Korean adults was approximately 86.2% in 2013, and is continuously growing. Moreover, the Internet use rate among young people in their twenties and thirties was approximately 99.7%. Furthermore, according to Google Korea’s marketing research on smartphones, smartphone penetration in South Korea reached 73% in July 2013, placing Korea at number one [16]. Notably, approximately 92% of the younger generation in their twenties through thirties own smart devices in Korea [15]. Overall, the findings from this study will help scholars and practitioners widen their comprehension of health-app use.

### Theoretical Backgrounds and Hypothesis Building

Although there exist numerous cognitive factors that can potentially stimulate people to use health apps, we paid considerable attention to the following four main factors: (1) health consciousness, (2) health information orientation, (3) eHealth literacy, and (4) health-app use efficacy. We selected these four factors by considering primarily the general functions of health apps. First, because the fundamental function of health apps is to manage one’s own health conditions [5], health consciousness is inherently related to health apps. Next, people use health apps in order to seek health information as well as to monitor their health conditions rather than to gain actual physical aid. This implies that health-app use is more relevant to health information-seeking behaviors. Thus, we focused mainly on health information orientation [17]. Third, it must be considered that such health information from health apps requires the competence of users to accurately comprehend the information accessed; this is known as the literacy of health information. Accordingly, paying attention to individuals’ abilities to decipher the meaning of Internet health information (eHealth literacy [17]), this study examined the role of eHealth literacy in health-app use. Furthermore, with regard to eHealth literacy, cognitive differences exist in individuals’ abilities to find and understand adequate health information [18-19]. This result implies the potential role of health-app use efficacy in mediating the relationship between eHealth literacy and health-app use. With this reasoning, we scrutinized the potential direct and indirect effects of the four cognitive factors on individuals’ actual use of health apps. This section will elaborate on each of these factors and propose multiple hypotheses for the study.

First, health consciousness basically refers to the extent to which a person takes care of his/her own health [17,19]. People with higher levels of health consciousness are more likely to have healthy habits, spend more time on exercise and healthy activities, actively gather health information from various sources, and avoid unhealthy situations [17,19]. In particular, such people are interested in seeking a diverse range of health information in order to gather more accurate information [20]. Moreover, previous research has demonstrated that health consciousness positively influences people’s information-seeking behaviors on the Internet [17]. Considering this influential role of health consciousness in health information-seeking behaviors, it is quite reasonable to expect that the more conscious a person is of his/her own health, the more actively she/he will use health apps. Based on this argument, this study established the following hypothesis (H1): Health consciousness will be positively associated with the extent of health-app use.

Health orientation is related to an individual’s proactive behaviors of taking care of his/her health condition [21]. Moorman and Matulich [22] defined health orientation as “a goal-directed arousal to engage in preventive health behaviors”. Specifically relevant to health-information seeking behaviors, Dutta-Bergman conceptualized health information orientation as “the extent to which the individual is willing to look for health information” [17]. More specifically, people with higher levels of health information orientation are more likely to gather health information from various sources. Moreover, Basu and Dutta stated that, “a high level of health-information orientation suggests the willingness to look for issues related to health and to find out ways to educate oneself about these issues, including the consumption of those communication channels that serve as potential sources of information regarding the issue” [19]. Considering the consumption of various channels for health information, health information orientation may be closely related to a person’s use of health apps as useful tools for seeking health information. Hence, this study established the following hypothesis (H2): Health information orientation will be positively associated with the extent of health-app use.

Previous research on health information-seeking behaviors has constantly argued the importance of literacy regarding health information from online sources, often referred to as eHealth literacy [5,18,23]. This is because the acquisition of more information does not necessarily mean better information. According to Norman and Skinner, eHealth literacy can be defined as “the ability to seek, find, understand, and appraise health information from electronic sources and apply the knowledge gained to addressing or solving a health problem” [18]. Based on this definition of eHealth literacy, it is comprehensible that when a person can better seek and understand online health information, she/he may be more motivated to use health apps as electronic sources. Thus, this study established the following hypothesis (H3): eHealth literacy will be positively associated with the extent of health-app use.

Related to eHealth literacy, we must consider that individuals have different levels of ability in using health apps. To better understand this notion, the concept of health information efficacy is useful. According to Basu and Dutta-Bergman, health information efficacy basically refers to “the perception of access to or the availability of health information resources” [19]. Furthermore, paying more attention to behavioral aspects, Yun and Park [24] proposed the concept of Internet health information use efficacy that is reliant on the concept of Bandura’s self-efficacy [25]. Here, self-efficacy is conceptualized as a person’s ability to achieve a directed goal. Thus, Internet health information use efficacy refers to the individual’s cognitive ability to strategically seek the necessary information by selectively using certain communicative channels. Based on these arguments regarding Internet health information use efficacy, this current study proposed the concept of health-app use efficacy, which is referred to as the cognitive ability to use health apps in order to access and seek health information.

Here, it is meaningful to focus on the potential relationship between eHealth literacy and health-app use efficacy. As stated above, eHealth literacy is closely related to an individual’s cognition of his/her own ability to seek and understand online health information [18,26]. Such self-efficacy related to online behaviors can be significantly associated with the use of mobile tools with online functions—more specifically, health apps on smart devices in this study. In other words, it is plausible that a person with higher levels of eHealth literacy is more likely to perceive that she/he has a better ability to use health apps. This belief implies the positive effect of eHealth literacy on health-app use efficacy. Ultimately, it also depicts that health-app use efficacy mediates the relationship between eHealth literacy and the extent of health-app use. Consequently, this study tested the following hypothesis (H4): Health-app use efficacy will positively mediate the relationship between eHealth literacy and the extent of health-app use.

For a more thorough analysis of the relationships among the main study variables, we differentiated between two different types of health-app users based on the nature of the health apps they use. In order to do this, we must first differentiate between two types of health-app uses: information-oriented use and behavior-oriented use. Information-oriented use refers to searching for health information (eg, symptoms, medication, preventive care) on apps. Behavior-oriented use involves active monitoring, recording, and management of health conditions through apps. Some examples of behavior-oriented use are using the app, “Diabetes in Check”, to record and monitor one’s daily insulin levels and calorie intake, or using the app, “Runkeeper”, to keep track of one’s daily fitness routine and history. Recognizing the different functional uses of health apps, the two types of health-app users that we identified are as follows: (1) information-oriented users, or single-purpose users, and (2) information-behavior users, or dual-purpose users. Ultimately, this study explored how the relationships among the five study variables would differ between these two groups of users. For this process, the following research question (RQ1) was explored: How will the relationships among the five study variables differ between single-purpose (information-oriented) users and dual-purpose (information-behavior) users?

## Methods

### Participants

For this present study, we used a subset of data collected for a larger research project, which examined Koreans’ general use of media for health information. The data were collected through an online survey administered by a Korean professional research company, well-known for managing the largest sampling pool in Korea. The sample for the larger project was chosen through a proportionate stratified sampling method, considering gender, age, and residential area. All survey participants were informed of the overall study goals and procedures. Only those who agreed to participate in the online survey were given access to the survey.

The questionnaire, conducted in Korean, included a question that asked the participants to report the different types of media they used in order to search for health information. Only data from those participants that marked the item of “mobile health apps” for this particular question were included in this current study. In other words, all participants included in the current study used health apps for information-oriented purposes.

Through this process, we were able to obtain a total of 765 surveys. There was a negligible difference in terms of gender composition: 50.6% (387/765) were male and 49.4% (378/765) were female. In terms of residential area, participants were from metropolitan areas (40.0%, 306/765), middle-size cities (36.3%, 278/765), and rural areas (23.7%, 181/765). All participants were adults whose ages ranged from 19 to 59 years; the average age was 37.1 years. In terms of educational attainment, the majority of participants held either a college degree (67.5%, 516/765) or a high school degree (22.7%, 249/765). About 9% (69/765) of the participants had graduate degrees (eg, MA or PhD).

In order to identify the different types of health-app users, those participants within our sample who further indicated the use of health apps for behavior-oriented purposes (use of health apps to monitor and manage their health conditions, such as blood pressure, blood sugar, history of exercise, etc) were categorized as information-behavior, dual-purpose users. The remaining participants were categorized as information-oriented, single-purpose users. A slightly larger portion of the survey participants (55.3%, 423/765) engaged in both information-oriented and behavior-oriented use, compared to those who engaged only in information-oriented use (44.7%, 342/765). In terms of the gender composition of the two groups, it was observed that, while there were slightly more female participants in the information-oriented use group (50.6%), there were slightly more male participants in the information-behavior use group (51.5%, 387/765). However, this gender difference was negligible for both groups. The average age of information-oriented users was slightly older (mean 38.9, SD 10.6) than that of information-behavior users (mean 35.7, SD 10.6).

### Instruments

#### Overview

All measures, except for the extent of health-app use, were constructed as 5-point Likert-type scales (eg, 1=strongly disagree, 5=strongly agree). Reliability tests for the four composite measures of this study reached acceptable Cronbach alphas (higher than .70).

#### Extent of Health-App Use

The extent of health-app use was conceptualized as the intensity of using health apps and was measured through a single item measured on a 6-point scale. Specifically, the participants were asked to answer the following question, “In the last month, how much time did you spend using health apps?” (1=less than 1 hour, 2=1-2 hours, 3=2-4 hours, 4=4-6 hours, 5=6-10 hours, and 6=more than 10 hours). As the extent of health-app use is a unidimensional factor, the use of a single-item measure is quite acceptable [27,28]. Moreover, in place of using the typical Likert-type options, such as a 5-point scale with options such as “much”, “very much”, and “little”, we created and used categories composed of six points, corresponding to the amount of time spent on health apps, in order to obtain a more objective and reliable measurement of the concept. According to previous research [29-31], this type of data format can be used for common parametric tests. Furthermore, following the guidelines from Kline [29] and Lee and Lim [32], the bootstrapping analysis was applied for the path analysis in order to eliminate any standard errors from the non-normal distribution.

#### Health Consciousness

This factor was measured through Dutta-Bergman’s scale [17], which measured the health-consciousness attitude through five items. Due to a low factor loading score (smaller than .50), one item was removed from further analysis. Examples of the items are as follows: “I am doing relatively well in taking care of my health” and “My health depends on how well I take care of myself”. The reliability score for this measurement was acceptable (mean 3.16, SD 0.64, N=765, alpha=.84).

#### Health Information Orientation

In order to measure health information orientation, we used Dutta-Bergman’s original scale [17] composed of eight items. In the process of the factor analysis, one item was removed from further analysis due to its low factor loading score. Example of the items are as follows: “To be and stay healthy, it is critical to be informed about health issues” and “When I take medicine, I try to get as much information as possible about its benefits and side effects”. This factor had an acceptable Cronbach alpha score (mean 3.48, SD 0.55, N=765, alpha=.86).

#### eHealth Literacy

In order to measure eHealth literacy, we used four items from Norman and Skinner’s scale [17]. Examples of these items are as follows: “I know how to find useful health information through the Internet” and “I have the skills I need to evaluate the health resources I find on the Internet”. The reliability score for this measurement was acceptable (mean 3.23, SD 0.59, N=765, alpha=.85).

#### Health-App Use Efficacy

In order to measure this variable, we reworded four items from Compeau and Higgins’ scale for computer self-efficacy [33]. Examples of those four items are as follows: “It is easy to learn how to use health apps on my smartphone” and “I can evaluate well the quality of health apps on my smartphone”. This factor also had an acceptable Cronbach alpha score (mean 3.23, SD 0.64, N=765, alpha=.87).

## Results

### Descriptive Statistics

Before conducting the path analysis, we analyzed the descriptive statistics for the five main variables. Through a series of independent samples *t* tests, one-way analysis of variance (ANOVA), and a bivariate correlation analysis, we checked for any differences in the variables in terms of gender, age, education level, and use patterns. First, we found significant gender differences in health consciousness (*M*_male_=3.30, *M*_female_=3.03, *t*=6.06, *P*<.001) and eHealth literacy (*M*_male_=3.31, *M*_female_=3.15, *t*=3.83, *P*<.001). Male participants reported higher scores for these two variables. Next, while age was positively correlated with health information orientation (*r*=.157, *P*<.001), it was negatively correlated with health-app use efficacy (*r*=−.136, *P*<.001) and the extent of health-app use (*r*=−.107, *P*<.001). Third, the results from ANOVAs reported significant educational differences in health consciousness (*F*_2,762_=5.20, *P*=.006) and eHealth literacy (*F*_2,762_=1.86, *P*=.019). The post-hoc tests for these two ANOVAs indicated that people with higher educational backgrounds reported higher levels of health consciousness and health-app use efficacy. Last, the results from the independent samples *t* test indicated that, except for health consciousness, the levels of all other four variables were significantly higher among information-behavior users compared to information-oriented users (see Table 1).

**Table 1:** Results for independent samples *t* test between information-oriented and information-behavior users.

Variable	Info-oriented	Info-behavior	*t* value	*P* value
	mean (SD)			
Health consciousness (HC)	3.12 (0.64)	3.20 (0.63)	−1.77	.08
Health information orientation (HIO)	3.38 (0.55)	3.60 (0.55)	−4.44	<.001
eHealth literacy (eHL)	3.12 (0.56)	3.32 (0.60)	−4.87	<.001
Health-app use efficacy (HAUE)	3.12 (0.68)	3.40 (0.67)	−5.85	<.001
Extent of health-app use (HAU)	2.84 (1.34)	3.43 (1.51)	−5.59	<.001

### Hypotheses Tests

**Figure 1:**
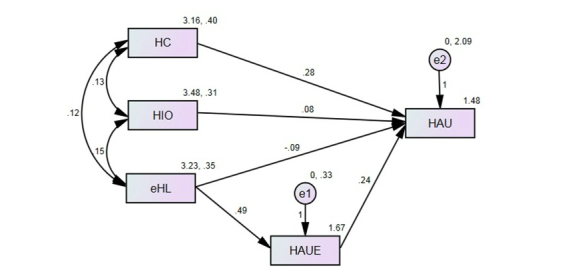
Initial path model of main study variables with entire sample. HC: Health Consciousness; HIO: Health Information Orientation; eHL: eHealth Literacy; HAUE: Health-App Use Efficacy; HAU: Extent of Health-App Use; e1: Standard Error for HAUE; e2: Standard Error for HAU.

**Figure 2:**
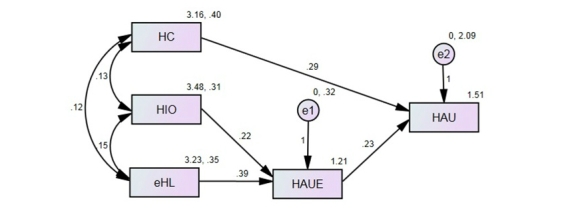
Final path model of main study variables with entire sample. HC: Health Consciousness; HIO: Health Information Orientation; eHL: eHealth Literacy; HAUE: Health-App Use Efficacy; HAU: Extent of Health-App Use; e1: Standard Error for HAUE; e2: Standard Error for HAU.

For testing the multiple hypotheses, we developed a path model composed of five paths. In order to test these hypotheses, we conducted a path analysis using AMOS 21 (SPSS software). Further, in order to minimize the standard errors from the non-normal distribution, we followed guidelines from Kline [29] and Lee and Lim [32] and conducted a bootstrapping analysis using a sub-sample of 200 from our study sample. Therefore, the *P* value for each path was calculated through a bias-corrected percentile method. We checked both the comparative and absolute fit indices in order to evaluate the goodness-of-fit of the proposed path model: comparative fit index (CFI; higher than .90), incremental fit index (IFI; higher than .90), and standardized root-mean squared residual (SRMR; lower than .10). Although the results from the path analysis of the initial model (see Figure 1) presented acceptable model fits (*χ* ^2^_2_= 27.5, CFI=.95, IFI=.95, SRMR=.04), the modification indices indicated the necessity to add a path from health information orientation to health-app use efficacy. To develop the final model, we removed two insignificant paths and added one path (see Figure 2). As a result, the final model illustrated much better model fits (*χ* ^2^_3_=1.02, CFI=1.0, IFI=1.0, SRMR=.007). Comparing the initial model to the final model, the chi-square largely and significantly decreased by 26.4 as the degree of freedom increased by one unit. H1 hypothesized a positive association between health consciousness and the extent of health-app use. Fully supporting H1, health consciousness positively and strongly impacted the use of health apps (beta=.286, *P*=.012).

H2 and H3 focused on the roles of health information orientation and eHealth literacy in directly influencing the extent of health-app use. With regard to these two hypotheses, the results from the path analysis indicated that neither health information orientation (beta=.08, *P*=.38) nor eHealth literacy (beta=−.09, *P*=.508) had a direct effect on the extent of health-app use (see Figure 1). These results indicate that H2 and H3 were rejected. However, as the final path model (Figure 2) indicates, health information orientation strongly impacted health-app use efficacy (beta=.220, *P*=.011). This reveals the indirect effect of health information orientation on the actual use of health apps. Therefore, in order to test the role of health-app use efficacy in mediating the relationship between health information orientation and the extent of health-app use, we used Sobel’s test. The test result found a significant mediating effect of health-app use efficacy (Sobel’s statistic=2.45, *P*=.014).

Next, paying attention to the influential cognitive role of self-efficacy in individuals’ actual behaviors, we focused on health-app use efficacy. In this study, we hypothesized that health-app use efficacy would positively mediate the relationship between eHealth literacy and extent of health-app use (H4). The results from the path analysis indicated that eHealth literacy strongly and positively affected health-app use efficacy (beta=.39, *P*=.005), which ultimately impacted the extent of health-app use (beta=.233, *P*=.023). Additionally, in order to test this mediating effect of health-app use efficacy, we used Sobel’s test. The result from Sobel’s test fully supported the mediating effect of health-app use efficacy (Sobel’s statistic=2.67, *P*=.007), thereby fully supporting H4.

Last, through RQ1, this study explored how the relationships among study variables would differ across the two groups of health-app users—information-oriented users vs information-behavior users. For this exploration, we conducted a multi-group structural equation modelling (SEM) for the final model (see Figure 2) and compared four pairs of regression coefficients for the two groups. Table 2 shows the results from the statistical comparison. The results indicated that only the path from health-app use efficacy to the extent of health-app use was statistically different between the two groups (*Z*=−2.14, *P*=.03). Specifically, while the direct effect of health-app use efficacy on the extent of health-app use was statistically significant among information-behavior users (beta=.319, *P*=.008), such effect was not significant among information-oriented users (beta=−.045, *P*=.734).

**Table 2:** Comparisons of regression coefficients between information-oriented users and information-behavior users.

Path	Information-oriented users, *β*	Information-behavior users, *β*	*Z* score	*P* value
HC^a^ → HAU^e^	.193	.315	−.73	>.10
HIO^b^ → HAUE^d^	.189	.221	−.31	>.10
eHL^c^ → HAUE	.352	.42	−.77	>.10
HAUE → HAU	−.045	.319	−2.14	.03

^a^HC: health consciousness

^b^HIO: health information orientation

^c^eHL: eHealth literacy

^d^HAUE: health-app use efficacy

^e^HAU: extent of health-app use

## Discussion

### Principal Findings

Considering the lack of studies on the motivational factors of health-app use, the main goal of this study was to explore how cognitive factors would motivate individuals’ use of health apps. In particular, paying attention to two different types of health-app use—information-oriented (single-purpose) use and information-behavior (dual-purpose) use—we focused on four cognitive factors: health consciousness, health information orientation, eHealth literacy, and health-app use efficacy. Before conducting the main path analysis, we checked for differences in the five main variables across gender, age, education, and patterns of health-app use. Consistent with the previous research on health apps [5], younger participants reported higher scores for both health-app use efficacy and extent of health-app use; further, participants with higher educational backgrounds tended to be more conscious of their health and have higher levels of eHealth literacy. Moreover, this study found that, compared to women, men also reported higher levels of health consciousness and eHealth literacy. These results support the findings of the previous research. Another notable finding is that information-behavior users reported higher scores for most variables compared to information-oriented users.

In addition to these descriptive findings, a number of meaningful findings were observed through a path analysis. First, supporting the findings of previous research regarding the positive functions of health consciousness [17], health consciousness in this study was also found to be positively and significantly associated with individuals’ use of health apps. That is, individuals more interested in taking care of themselves are more likely to use health apps than individuals less conscious of their health. This finding reaffirms the existing knowledge that understands health consciousness to be one of the most dominant factors guiding the adoption of health technologies.

Next, it is noteworthy that, unlike the initial predictions, there was no direct effect of health information orientation and eHealth literacy on the extent of health-app use. Rather, the effects of these two factors were mediated by health-app use efficacy. This indicates the significant roles of health-app use efficacy. In general, people who are more oriented toward actively seeking health information and having a better understanding of online health information tend to be more efficient in using health apps as well as allocating more time for health apps. However, as the results from the multi-group SEM show, the effect of health-app use efficacy on the extent of health-app use was statistically significant only among information-behavior users. This may be due to the displacement of media for health information [34,35]. Information-oriented users may irregularly and occasionally use health apps only at those times that they are in need of certain types of health information. On the other hand, information-behavior users also tend to occasionally seek health information, but manage their health on a regular basis. Here, it must be considered that general health information can be obtained through various online sources. This implies that health apps for general health information can be more easily displaced by these convenient alternatives. However, considering their habitual use of health apps for health management, information-behavior users may need to invest in additional resources in order to seek and routinize alternative media. Moreover, when a person is efficiently using a certain health app on a regular basis, she/he will be more inclined to continue using the app and more reluctant to displace it. This particular finding addresses the key roles of health-app use patterns for determining the intensity/extent of health-app use and, further, theoretically contributes to widening our understanding of the behavioral aspects of health-app use.

Furthermore, this significant role of health-app use efficacy suggests the following practical implication. Health-app use efficacy is conceptually reliant on Bandura’s [25] concept of self-efficacy. Moreover, with regard to technology use, self-efficacy is often related to the perceived ease of using certain technologies. Consequently, when an individual perceives higher ease of using health apps, she/he may feel higher health-app use efficacy. This addresses the importance of creating health apps that allow higher levels of ease and convenience in use. Although popular health apps provide users with detailed, useful information, many of them require users to complete multiple steps in order to access such information. For example, in order to obtain information about one’s daily calorie intake through the smartphone app, “Lose It”, users must first complete several steps and provide many details about their meals (eg, exact categories and amounts of each component of their breakfast, lunch, dinner, and snacks). Although this app provides users with accurate information, the repetitive and complex process requires users to invest a great amount of time and mental energy. This may negatively impact users’ health-app use efficacy, ultimately affecting their willingness to use the app. Therefore, there is a need for practitioners to work on the simplification and reduction of algorithms in constructing health app processes.

### Limitations

Overall, these findings will serve as helpful empirical and theoretical foundations for future research on health apps. Moreover, they may guide practitioners in developing more realistic and strategic plans to enhance health-app consumption. Nevertheless, considering the limitations of this present study, the following points are recommended for future research. First, as stated above, people use health apps for different reasons—to exercise, to lose weight, to check blood sugar, to track period cycles, and so forth. Based on the above uses and the gratification theory [36], the different purposes for using health apps may be related to motivational factors. Although this present study focused on two different general patterns of health-app use—information-oriented use and information-behavior use—future research will benefit from a more thorough exploration of the more diverse range of functions that health apps have, particularly the functions of those that focus on specific types of health conditions and needs (eg, apps for Type 2 diabetes, Alzheimer’s, pregnancy, fitness). Moreover, it is possible that the time spent on health apps may be determined by the specific functions afforded by the health apps. For instance, the use of health apps to check for blood sugar levels will require much shorter amounts of time than the use of fitness apps that track how many miles one has been running; although it is possible that the former type of apps may be used on more frequent levels. Consequently, for future research, it is recommended to grant attention to the multiple aspects of health-app use, particularly with regard to the extent and frequency of use, based on a more detailed identification of the specific features and functions of various apps.

Next, another limitation of this current study is the relatively large portion of participants with college degrees. Although it has been often observed that individuals with higher levels of education tend to more actively adopt new technologies (eg, smart devices) [37], it is still necessary to collect more representative samples for future research. Considering the effects of social influences on technology adoption and use [36,38-40], it becomes more vital to collect samples with higher representativeness with regard to the socioeconomic status (SES). This is mainly because social influences are closely connected to educational levels and SES. That is, individuals with higher educational levels and SES are more likely to be affected by the subjective norms of their influential others who are more open to new technologies. Accordingly, it is recommended for future research to further consider the roles of educational levels and SES that are related to social influences by collecting more representative samples.

Finally, in terms of health information-seeking behaviors, we need to consider the following points. As Baumgartner and Hartmann [37] argued, searching for online health information is closely related to one’s level of health anxiety. Moreover, research depending on information management theory [41-43] has stated that a person diagnosed with a chronic illness (eg, AIDS, cancer) will want to manage the amount of available information they are exposed to, rather than proactively seek information in order to reduce uncertainty. These findings commonly indicate that information-seeking behaviors through health apps are possibly moderated by people’s actual health conditions. In other words, it is possible that people with chronic illnesses are less inclined to seek further information even though they have high levels of eHealth literacy as well as health-app use efficacy. Therefore, future research may consider further studying health-app use in relation to individuals’ personal health conditions.

### Conclusions

As a specific realm of mobile health, smartphone health apps are a significant form of technology that people have become increasingly interested in. However, we have had little understanding of the motivational factors that guide people to use health apps. Accordingly, this study aimed at exploring the effects of four cognitive factors—health consciousness, health information orientation, eHealth literacy, and health-app use efficacy—on the extent of health-app use. The results from a path analysis addressed the significant direct effect of health consciousness as well as strong mediating effects of health-app use efficacy. These findings contribute to broadening our comprehension of the new, digital dimensions of health management that revolve in particular around mobile technology.

## Abbreviations

CFI: comparative fit index
eHL: eHealth literacy
HAU: extent of health-app use
HAUE: health-app use efficacy
HC: health consciousness
HIO: health information orientation
IFI: incremental fit index
SEM: structural equation modeling
SES: socioeconomic status
SRMR: standardized root-mean squared residual
